# Isolated saccular aneurysm of the external jugular vein

**DOI:** 10.4322/acr.2020.188

**Published:** 2020-12-14

**Authors:** Hari Janardanan Pillai, Nilanjan Roy, Pankaj Purushotam Rao, Khushdeep Kaur Shergill, Divya Shelly, Basil Badarudeen

**Affiliations:** 1 Armed Forces Medical College, Department of Surgery, Pune, Maharashtra, India; 2 Indian Navy Hospital Ship Asvini, Mumbai, Maharashtra, India

**Keywords:** Aneurysm, Jugular Veins, Venous Thrombosis

## Abstract

Venous aneurysm of the head and neck is a rare clinical entity due to its asymptomatic nature and tendency of clinicians to report only surgical results. Whereas the primary aneurysm of internal jugular vein (IJV) in children is being increasingly recognized, secondary aneurysms of veins of the head and neck in adults, notably the external jugular vein (EJV) aneurysm remains only in anecdotal case reports. We present the case of a 63-year-old previously healthy woman who presented with a gradually progressive right lateral neck swelling over the last 18 months. Following the evaluation, she was diagnosed as a case of isolated spontaneous right-sided EJV aneurysm and was managed by surgical excision of the aneurysm.

## CASE REPORT

**Figure 1:**
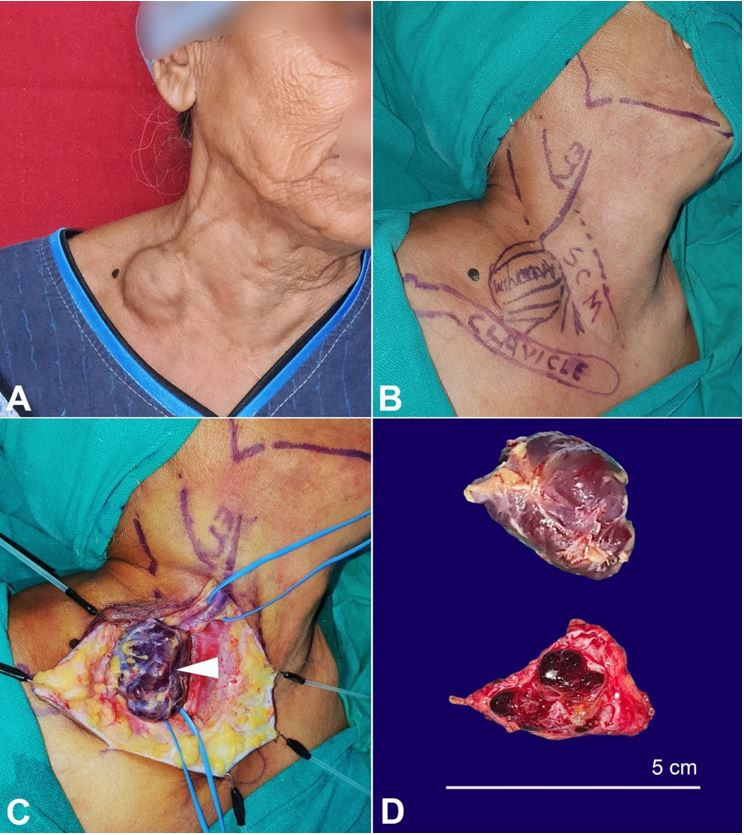
**A –** Gross examination of the neck region with a 5-cm swelling over right supraclavicular region; **B –** preoperative marking of the patient after draping in the OR, SCM – sternocleidomastoid muscle, Clavicle – Right Clavicle, EJV - Right EJV; **C –** intraoperative image. Note the saccular aneurysm along the looping of the right EJV (arrowhead); **D –** Gross view of the excised specimen of aneurysm (upper image) and the cut surface of the opened specimen showing intraluminal thrombus (lower image).

**Figure 2:**
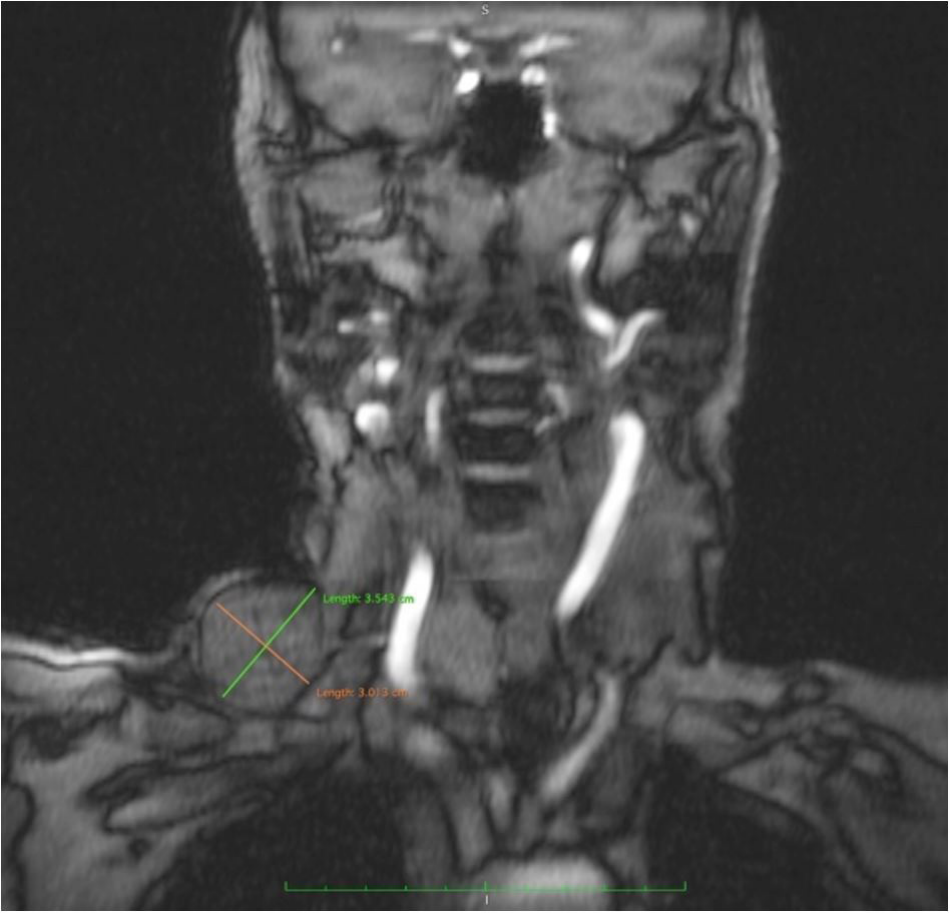
MR venogram of the neck (T1 weighted image) showing focal outpouching arising from the anterolateral aspect of the right EJV, immediately cranial to its confluence with the right subclavian vein, measuring 3.5 × 3.01 cm.

A 63-year-old, previously healthy lady, presented with complaints of insidious-onset swelling over the right side of her neck since18 months, which gradually progressed from the initial size of 2 cm to 5 cm at presentation. She also noted an increase in the size of the swelling during straining and coughing. Though the swelling was initially painless, she experienced a vague, dull aching pain in the swelling in the last couple of months, which forced her to seek medical evaluation. There was no other contributory history of trauma or surgical intervention in the neck. On evaluation, she had a non-tender, soft, and non-pulsatile swelling in her right supraclavicular region, measuring 5 cm x 4cm (Figure 1A, B). The swelling was reducible and enlarged in size with the Valsalva maneuver. The ultrasonography with color Doppler of her neck revealed a 3.5 cm lesion with no internal vascularity concerning the right EJV. Magnetic Resonance Venogram (MRV) reported a focal outpouching arising from the anterolateral aspect of right EJV immediately cranial to its confluence with right subclavian vein measuring 3.3 x 3.4 x 3.7 cm with an intraluminal thrombus (Figure 2). The remaining vasculature of the neck appeared normal. She was diagnosed as a case of an isolated saccular aneurysm of the right EJV. Given the recent onset pain and progressive increase in size, she underwent exploration of the right side of the neck, which revealed a 4x4 cm saccular aneurysm of the proximal right EJV with intraluminal thrombus (Figure 1C and D).

Proximal and distal control of EJV were obtained, and the aneurysm was excised with ligation of the right EJV. The patient had an uneventful postoperative recovery and was discharged on postoperative day 2.

Histopathology of the specimen was consistent with a true aneurysm of the vein. The transverse section of the aneurysm showed an abnormally dilated venous lumen with thrombus, surrounded externally by a thinned tunica media (H&E stain x 100). Elastic

**Figure 3:**
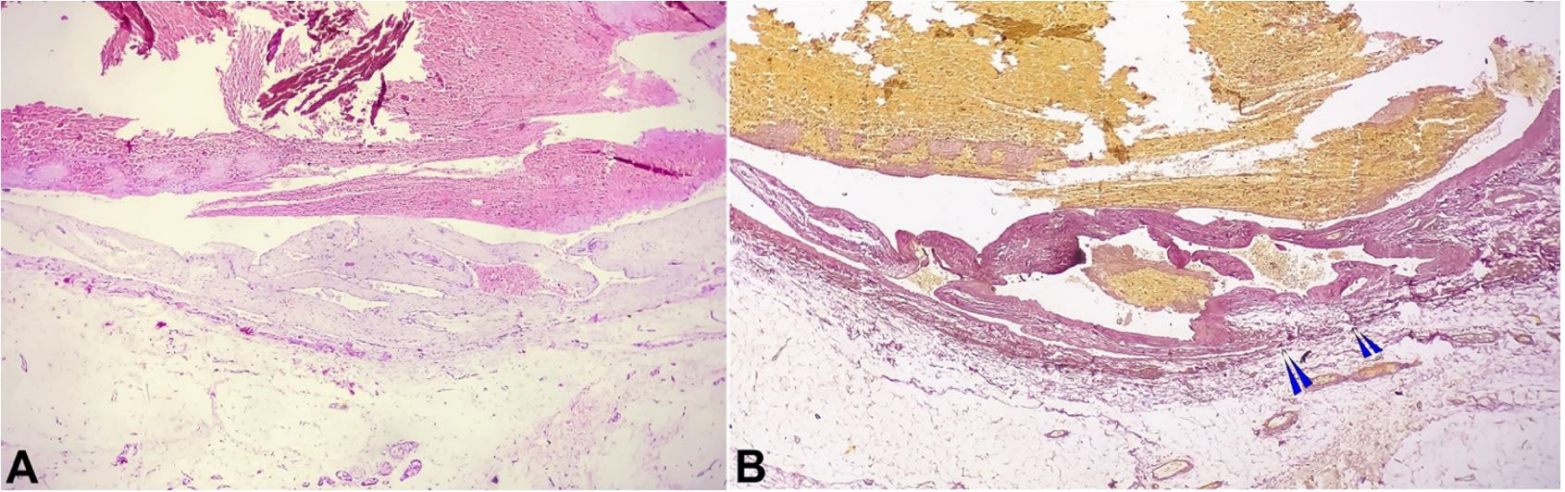
Photomicrograph of the surgical specimen showing the complete loss of the elastic fibers in the vessel wall (multiple arrowheads); **A –** (H&E, 100X) and **B –** (EVG stain, 100X).

Van Gieson (EVG) stain revealed a complete loss of elastic fibers in the vessel wall (Figure 3). Her follow up visit at one month revealed a well-healed scar in the neck with no complaints.

## DISCUSSION

Venous aneurysms in the head and neck are rarely encountered in clinical practice owing to the low-pressure system of the superior vena cava.^1^ ^-^ ^4^ The majority of them involve the internal jugular vein (IJV), followed by the EJV and the anterior veins in decreasing frequency.^5^ They can either be congenital or acquired. Congenital aneurysms are fusiform dilatations of the vein, commonly involving IJV, and are supposed to be a consequence of the weakness of the elastic layers and muscle cells.^2^ ^,^ ^3^ ^,^ ^6^ The acquired aneurysms are typically saccular, occur spontaneously or secondary to trauma, surgical intervention or diseases involving veins like endophlebosclerosis and endophlebohypertrophy.^7^ ^,^ ^8^

EJV aneurysm, by far, is exceedingly rare, and its exact incidence is still not known.^9^ EJV aneurysm is commonly found in elderly males, on the right side. The risk factors for their development include trauma, thoracic outlet syndrome, tumors, hormonal therapy, or increased pressure in the vena cava system.^3^ ^-^ ^5^ They are commonly saccular and rarely fusiform.^3^ The majority of the patients present with an asymptomatic, soft, non- tender compressible swelling in the lateral aspect of the neck, which classically shows an increase in size on straining or with Valsalva maneuver in the absence of any bruit. Some patients may complain of dull aching or, more commonly, a “discomfort” or feeling of tightness over the swelling.^10^ The differential diagnosis includes (i) laryngocele, (ii) cavernous hemangioma, (iii) pharyngeal pouch, (iv) external laryngeal diverticulum, (v) superior mediastinal cyst, and (vi) cervical arterial, venous aneurysms or veno-lymphatic malformations.^10^ ^,^ ^11^ Noninvasive diagnostic imaging modality like doppler ultrasonography of the neck veins confirms the diagnosis.^12^ Further, CT angiography with digital subtraction angiography and MR venography can help to delineate the anatomical extent of the aneurysm, presence of feeder vessels, intraluminal thrombus besides aiding in pre-operative planning,^9^ ^,^ ^10^ ^,^ ^13^ EJV aneurysms rarely give rise to complications like thrombophlebitis, rupture, thrombus formation secondary to trauma, or pulmonary embolism.^14^ ^,^ ^15^

The indications for treatment are primarily cosmetic and rarely due to complications of the aneurysm per se.^16^ Clinical progression of EJV aneurysm is insidious and protracted without significant morphological transformations. Asymptomatic patients can be reassured and conservatively managed with watchful waiting.^2^ ^,^ ^9^ ^,^ ^14^ ^,^ ^16^ ^,^ ^17^ The management modalities for an aesthetically disfiguring and complicated aneurysm include surgical excision or endovascular coil embolization.^17^ The treatment of choice for saccular aneurysms of the EJV is complete surgical excision without venous reconstruction through a longitudinal, lateral neck incision under general or local anesthesia with minimal intraoperative and postoperative complications. Intravenous coil embolization, along with percutaneous injection of sclerosant foam, has recently emerged as a minimally invasive alternative.^9^ ^,^ ^13^ Though there is no evidence, at present, to prove its clinical efficacy compared to standard surgical excision, it offers a superior cosmetic outcome with lesser hospital stay at the expense of incurring higher treatment costs.

## CONCLUSION

Aneurysms of the EJV can occur spontaneously or as a sequel to trauma, surgical intervention, or diseases affecting the vein walls. It can be asymptomatic or can present with complications like thrombosis or inflammation. Clinical examination augmented by non-invasive color doppler ultrasonography confirms the diagnosis. Management depends on the symptoms complex of the patient. Whereas asymptomatic patients can be offered watchful waiting, symptomatic patients have to be managed with either surgical excision or endovascular embolization with minimal postoperative morbidity.

## Notes

**Aos autores:**

[[Q1: Insert a short statement indicating where was the study was carried out, including institution name, city, state and country. Q1]]
